# Severe Gallbladder Dyskinesia With 2% Ejection Fraction: A Comprehensive Clinicoradiologic and Pathologic Case Correlation

**DOI:** 10.7759/cureus.104416

**Published:** 2026-02-27

**Authors:** Newton Rahming, Shinelle De Almeida, Ulrica Armbrister, Stephan Corcho, Frederick Tiesenga

**Affiliations:** 1 Surgery, Caribbean Medical University, Willemstad, CUW; 2 Emergency Medicine, All Saints University School oF Medicine, Roseau, DMA; 3 Surgery, Windsor University School of Medicine, Canyon, KNA; 4 Surgery, St George's University, St George's, GRD; 5 General Surgery, West Suburban Medical Center, Chicago, USA

**Keywords:** biliary dysmotility, cholecystectomy, chronic cholecystitis, functional biliary disorder, gallbladder dyskinesia, hida scan

## Abstract

Gallbladder dyskinesia (GBD) is a functional biliary disorder characterized by biliary colic in the absence of cholelithiasis or mechanical obstruction and is identified by reduced gallbladder ejection fraction (EF) on cholecystokinin (CCK)-stimulated hepatobiliary scintigraphy. Patients may experience significant symptoms due to impaired gallbladder contractility and bile stasis despite the absence of gallstones or inflammatory structural abnormalities on imaging.

We present a 42-year-old male with a three-month history of episodic right upper quadrant (RUQ) pain triggered by fatty meals. Abdominal ultrasound demonstrated no cholelithiasis, sludge, wall thickening, or pericholecystic fluid. Contrast-enhanced computed tomography (CT) revealed a distended gallbladder without obstructing gallstones or inflammatory changes. CCK-hepatobiliary iminodiacetic acid (HIDA) scintigraphy demonstrated a markedly reduced gallbladder EF of 2%, consistent with severe contractile dysfunction. The patient underwent an uncomplicated laparoscopic cholecystectomy. Histopathology revealed mild chronic cholecystitis and cholesterolosis. At follow-up, he reported complete resolution of symptoms and tolerance of a normal diet.

This case highlights the diagnostic value of functional biliary imaging in symptomatic patients without gallstones or inflammatory structural abnormalities. Profound reduction in gallbladder EF may correlate with severe biliary dysmotility and favorable postoperative outcomes.

## Introduction

Gallbladder dyskinesia (GBD), classified under functional gallbladder disorders in the Rome IV criteria, results from impaired gallbladder contractility leading to biliary colic in the absence of cholelithiasis or mechanical obstruction, with symptoms occurring exclusively postprandially, particularly after fatty meals, and absent at rest [1]. Diagnosis requires exclusion of structural disease and demonstration of reduced gallbladder ejection fraction on cholecystokinin-stimulated hepatobiliary iminodiacetic acid (CCK-HIDA) scintigraphy [2]. The pathophysiology stems from impaired gallbladder contractility, resulting in reduced or absent bile release triggered by meals, which contributes to postprandial symptoms [3]. Patients clinically present with epigastric or right upper quadrant (RUQ) pain resembling classic biliary colic [3]. The pathophysiology of biliary dyskinesia is not fully understood but is thought to involve dysregulation of neurohormonal signaling, particularly abnormalities in CCK-mediated pathways that impair coordinated gallbladder contraction and bile secretion [4].

Laparoscopic cholecystectomy has become the standard treatment for symptomatic biliary dyskinesia [5]. The condition is diagnosed disproportionately in the United States, although its true prevalence is difficult to determine due to variability in diagnostic practices. GBD is estimated to account for a meaningful proportion of patients presenting with biliary colic but normal abdominal ultrasound findings, and it is most commonly diagnosed in younger individuals [5].

Magnetic resonance cholangiopancreatography (MRCP) may be considered when biliary obstruction or choledocholithiasis is suspected; however, in patients with normal ultrasound and computed tomography (CT) imaging and no biochemical evidence of obstruction, functional assessment via CCK‑HIDA scintigraphy remains the diagnostic modality of choice, as the condition is increasingly recognized yet remains diagnostically challenging because routine imaging, including ultrasound and CT, often appears normal [3,5]. Clinical evidence supports the use of HIDA scanning with CCK stimulation as the primary diagnostic tool for evaluating gallbladder motility. A severely decreased gallbladder ejection fraction (EF) on CCK-HIDA scintigraphy has been shown to correlate with gallbladder dysfunction and predict symptomatic improvement following cholecystectomy [6]. Evidence suggests that patients with significantly reduced EF, particularly <10%-15%, have the highest likelihood of symptom resolution after surgery [7]. To date, no pharmacologic therapy has demonstrated consistent or durable efficacy in the treatment of GBD. In patients with persistent biliary colic and markedly reduced gallbladder EF, laparoscopic cholecystectomy remains the only intervention associated with sustained symptom resolution [5].

This report presents a case of severe GBD with a strikingly low EF of 2%, normal mechanical imaging, and pathology demonstrating chronic inflammatory changes. The case provides a complete clinicoradiologic and pathologic correlation, contributing to the growing literature supporting surgical intervention in patients with severe dysmotility.

## Case presentation

A 42-year-old male presented with a three-month history of episodic RUQ abdominal pain radiating to the back; symptoms were consistently triggered by fatty meals, including fried foods and high-fat dairy products. The pain was colicky in nature and accompanied by nausea and occasional vomiting. He denied fever, jaundice, pruritus, changes in stool color, or weight loss. His medical history included controlled hypertension and asthma. Social history was notable for former tobacco use and occasional alcohol consumption.

On physical examination, he was afebrile with mild tachycardia (104 beats per minute (bpm)) but otherwise hemodynamically stable. Abdominal examination revealed mild RUQ tenderness without guarding, rebound, or a positive Murphy’s sign.

Laboratory evaluation outlined in Table 1 revealed mild anemia and mild electrolyte abnormalities, including hyponatremia and hypokalemia, both of which were corrected prior to operative intervention.

**Table 1:** Summary of relevant preoperative laboratory and hepatobiliary iminodiacetic acid scintigraphy results

Parameter	Results	Reference Range	Clinical Interpretation
Hemoglobin	12.3 g/dL	13.5 - 17.5 g/dL	Mildly Low
Aspartate Aminotransferase	28 U/L	10 - 40 U/L	Normal
Alanine Aminotransferase	28 U/L	7 - 56 \U/L	Normal
Alkaline Phosphatase	72 U/L	44 - 147 U/L	Normal
Total Bilirubin	0.6 mg/dL	0.3 - 1.2 mg/dL	Normal
Lipase	57 U/L	10 - 140 U/L	Normal
Sodium	134 mEq/L	135 - 145 mEq/L	Mildly Low (Corrected)
Potassium	3.3 mEq/L	3.5 - 5.1 mEq/L	Mildly Low (Corrected)

Liver function tests, including aspartate aminotransferase, alanine aminotransferase, alkaline phosphatase, and total bilirubin, were within normal limits. Pancreatic enzyme levels were also normal. 

CCK-HIDA scintigraphy was performed as a targeted functional imaging study following exclusion of structural pathology. It is not a routine biochemical test but a specialized nuclear medicine study indicated in persistent biliary-type pain with normal structural imaging.

An abdominal ultrasound demonstrated a normal gallbladder without cholelithiasis, sludge, wall thickening, or pericholecystic fluid. The common bile duct measured within normal limits. These findings effectively excluded acute or chronic calculous disease. 

**Figure 1:**
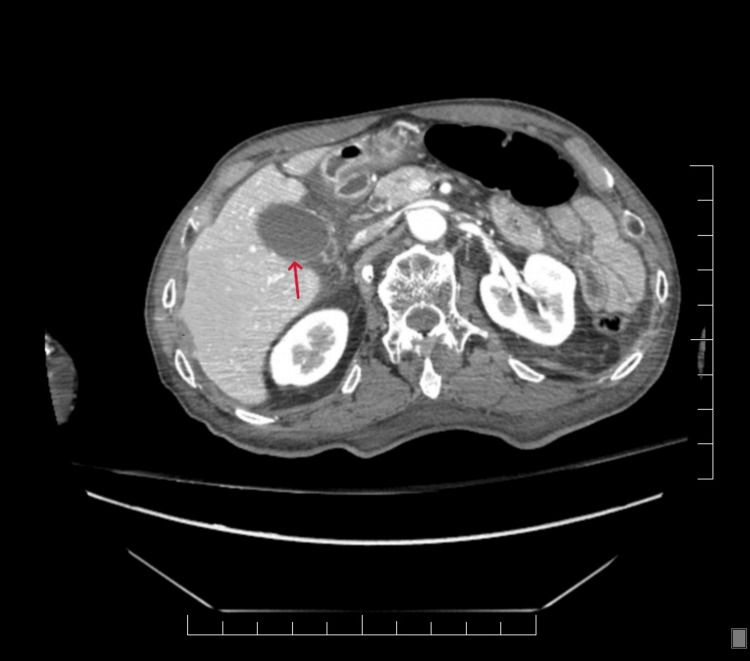
Abdominal computed tomography (CT) imaging Axial contrast-enhanced CT demonstrating a distended gallbladder (red arrow) without gallstones, wall thickening, or pericholecystic fluid.

Contrast-enhanced CT of the abdomen (Figure 1) demonstrated a distended gallbladder without wall thickening, pericholecystic fluid, radiopaque gallstones, or biliary ductal dilation. The gallbladder distension was consistent with impaired emptying rather than mechanical obstruction. The liver, pancreas, and surrounding structures were unremarkable. These findings further excluded structural or inflammatory hepatobiliary pathology and supported a functional etiology for the patient’s symptoms. 

The official ultrasound report documented a distended gallbladder without cholelithiasis.

Given persistent biliary colic symptoms with a distended gallbladder on imaging, CCK-HIDA scintigraphy was performed.

**Figure 2:**
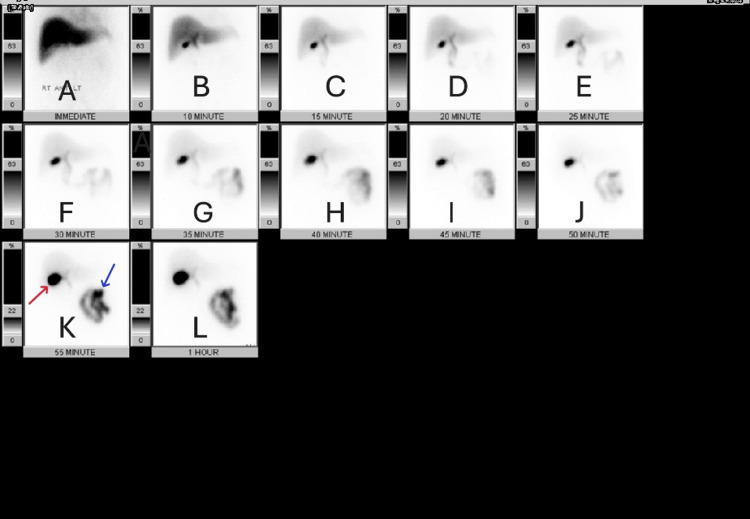
Dynamic hepatobiliary scintigraphy demonstrated impaired gallbladder filling and contraction Hepatobiliary scintigraphy study demonstrated normal hepatic uptake and prompt biliary excretion with tracer passage into the small bowel (Frames A-L); the gallbladder (red arrow); the small intestine (blue arrow); and Frames K and L showed no contraction of the gallbladder.

Dynamic imaging demonstrated normal hepatic tracer uptake and biliary excretion with tracer passage into the small bowel, confirming cystic duct patency (Figure 2, frames A-L). The gallbladder demonstrated minimal filling and no appreciable contraction following CCK administration (Figure 2, frames K-L).

**Figure 3:**
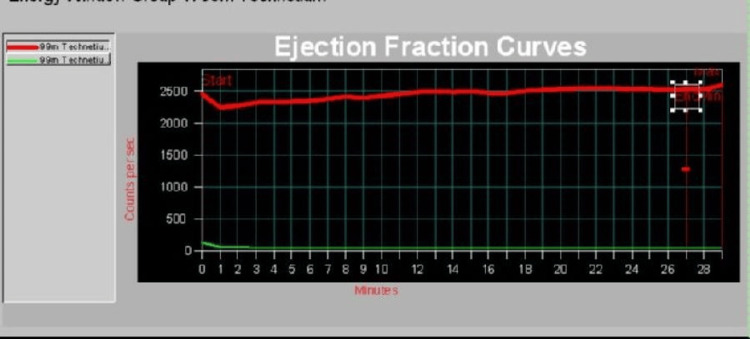
Gallbladder ejection fraction curve demonstrated absent contractile response The hepatobiliary iminodiacetic acid scan demonstrated normal hepatic uptake and tracer delivery (red line) with a flat gallbladder time–activity curve (green line) following stimulation. The (white square) on the right margin represents the imaging field calibration marker and does not reflect pathology.

Quantitative time-activity analysis curves demonstrated tracer kinetics following administration of 99mTc-mebrofenin. The upper curve represented background hepatic and biliary system activity, showing stable tracer uptake and excretion. The lower curve represented gallbladder activity and demonstrates no significant decline following stimulation, indicating absent gallbladder contraction. The calculated gallbladder EF was 2%, consistent with severe GBD (Figure 3).

After exclusion of structural pathology and confirmation of severe gallbladder dysfunction on CCK-HIDA scintigraphy, laparoscopic cholecystectomy was recommended for definitive treatment.

**Figure 4:**
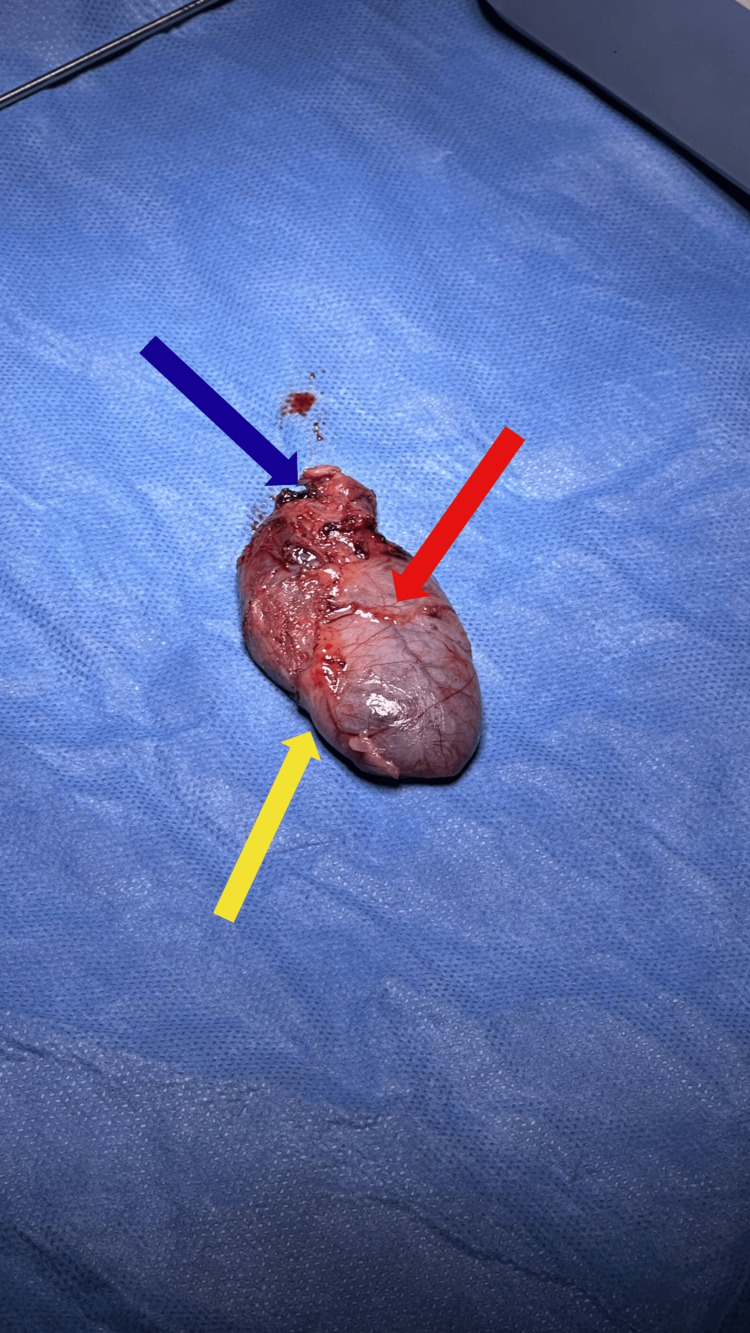
Gross specimen demonstrated a distended gallbladder without cholelithiasis Gallbladder pathology showed serosal vascular congestion (red), inflammatory changes at the neck (blue), and focal fibrotic wall thickening consistent with chronic disease (yellow).

Gross examination revealed a distended gallbladder with a smooth but hypervascular serosal surface and focal areas of congestion near the infundibulum, without gallstones or perforation (Figure 4). These findings were consistent with chronic inflammatory changes and correlated with prolonged functional bile stasis.

The patient underwent an uncomplicated laparoscopic cholecystectomy in September 2025 with minimal blood loss. 

**Figure 5:**
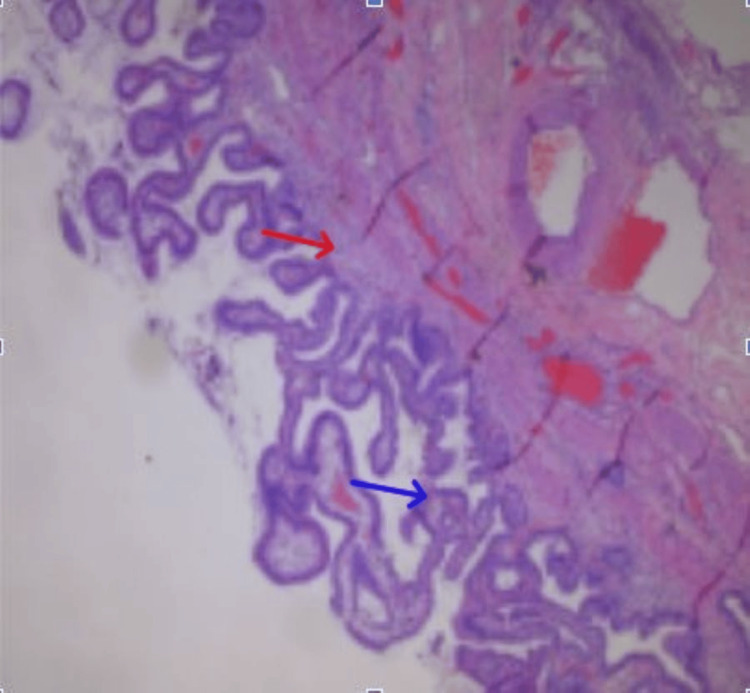
Histologic section demonstrated chronic cholecystitis and cholesterolosis (H&E stain) Microscopic examination of the gallbladder wall (H&E stain) demonstrated mucosal changes consistent with cholesterolosis (blue arrow) and transmural fibrotic and muscular hypertrophy consistent with chronic cholecystitis (red arrow).

Histopathologic examination of the resected gallbladder revealed mild chronic cholecystitis and cholesterolosis, characterized by lymphocytic infiltration of the lamina propria and foamy macrophages within the mucosa (Figure 5). No gallstones, dysplasia, or malignancy were identified.

The patient's postoperative course was uneventful. He resumed oral intake on postoperative day one and was discharged home on postoperative day two. At outpatient follow-up, the patient reported complete resolution of abdominal pain and tolerance of a normal diet. At the six-week follow-up, he remained asymptomatic with no recurrence of gastrointestinal symptoms.

## Discussion

GBD is a functional biliary disorder characterized by impaired gallbladder emptying in the absence of mechanical obstruction. Although historically considered a diagnosis of exclusion, it is now recognized as a distinct clinical entity and accounts for approximately 5%-10% of cholecystectomies performed in adults [5]. Gallbladder distension in the absence of gallstones, ductal dilation, or wall thickening supports impaired contractility rather than mechanical obstruction. In functional gallbladder disorders, distension reflects ineffective emptying and chronic bile stasis rather than structural blockage. Patients consequently present with classic biliary colic despite normal ultrasound or CT imaging, which often leads to diagnostic uncertainty and delayed management [5]. 

In this context, CCK-HIDA scintigraphy plays a central diagnostic role by providing an objective assessment of gallbladder contractility. An EF below 35% is widely accepted as abnormal, and surgical outcomes correlate strongly with the severity of dysfunction. Patients with markedly reduced EFs, particularly <10%, consistently demonstrate the highest rates of postoperative symptom resolution [6]. The present case, with an EF of 2%, reinforces the predictive value of severely reduced gallbladder contractility for favorable surgical outcomes.

The pathogenesis of GBD remains incompletely understood and is likely multifactorial. Proposed mechanisms include autonomic nervous system dysfunction, abnormal CCK receptor signaling, impaired smooth muscle responsiveness, and disrupted neurohormonal coordination within the biliary system [7]. A comprehensive evaluation is required to exclude alternative causes of biliary colic, including structural, inflammatory, and functional gastrointestinal etiologies [8]. Regardless of the initiating mechanism, impaired gallbladder contractility results in chronic bile stasis, which may promote mucosal irritation, epithelial dysfunction, and low-grade inflammation over time [4,9].

In this case, the histopathologic findings of mild chronic cholecystitis and cholesterolosis provide clinicopathologic correlation supporting progression from functional dysmotility to chronic inflammatory structural changes.

Although GBD is considered a functional motility disorder, impaired emptying can lead to sustained bile stasis, which promotes mucosal irritation, epithelial injury, and activation of inflammatory pathways within the gallbladder wall [10]. The presence of foamy macrophages characteristic of cholesterolosis supports the hypothesis that impaired motility alters bile composition and promotes lipid accumulation within the mucosa [10]. 

This concordance between clinical presentation, functional imaging, and histopathology strengthens diagnostic confidence and underscores the value of an integrated clinicoradiologic pathologic approach. Such correlation supports timely surgical intervention, as persistent functional impairment may contribute to chronic inflammatory structural changes.

A comprehensive evaluation is required to exclude alternative causes of biliary-type pain. In this case, considerations included acalculous cholecystitis, peptic ulcer disease, sphincter of Oddi dysfunction, and functional gastrointestinal disorders such as irritable bowel syndrome. Normal ultrasound and CT imaging effectively excluded cholelithiasis, biliary obstruction, and structural gallbladder inflammation [11]. Normal liver function tests, pancreatic enzymes, and the absence of systemic inflammatory markers further reduced the likelihood of acute hepatobiliary or pancreatic pathology [12]. 

While mild hepatic steatosis was incidentally observed, there is no established causal association between hepatic steatosis and GBD, and it rarely presents with isolated postprandial RUQ pain or biliary colic-like symptoms [13]. The exclusion of structural and inflammatory etiologies highlights the importance of functional assessment with HIDA scintigraphy in patients with persistent biliary-type symptoms.

Laparoscopic cholecystectomy remains the standard treatment for symptomatic GBD in patients who meet Rome IV criteria and demonstrate reduced gallbladder EF on CCK‑HIDA scintigraphy, where normal gallbladder EF values are typically ≥35%-40% and abnormal function is defined by values <35%, with severe reductions often <15-20%. Studies have demonstrated significant postoperative symptom improvement, particularly among patients with severely reduced EFs (<15%), where surgical intervention has been shown to provide significant symptom amelioration [6,14]. Patients with profoundly diminished gallbladder EFs, such as the 2% observed in this case, have been reported to experience some of the highest rates of durable postoperative symptom resolution among surgical cohorts with markedly reduced EFs [14].

Conservative measures, including dietary modification and pharmacologic therapy, may provide temporary symptom relief but are often insufficient due to the underlying motility disorder. Given the severity of dysfunction and reproducibility of symptoms in this patient, surgical intervention represented the most appropriate and definitive management strategy.

## Conclusions

This case highlights the importance of considering GBD in patients who present with biliary colic despite the absence of gallstones or inflammatory structural abnormalities. CCK‑stimulated HIDA scintigraphy remains essential for diagnosis by objectively assessing gallbladder contractility. In this patient, a profoundly reduced gallbladder EF of 2% aligned closely with the clinical presentation and predicted complete postoperative symptom resolution. Histopathologic findings of chronic cholecystitis and cholesterolosis illustrate that severe functional dysmotility may progress to structural inflammatory changes. Although inflammatory markers are not routinely required in the evaluation of GBD, they may help exclude acute inflammatory processes when clinically indicated. Early recognition and timely laparoscopic cholecystectomy can provide durable symptom relief by removing the dysfunctional gallbladder and eliminating the source of recurrent biliary pain.
